# Neue und wiederkehrende sexuell übertragbare Erreger: Nachwuchs- und Überlebenskünstler

**DOI:** 10.1007/s12326-021-00470-6

**Published:** 2021-11-02

**Authors:** Angelika Stary

**Affiliations:** Pilzambulatorien Wien, Universitätsklinik für Dermatologie der Medizinischen Universität Wien, Wien, Österreich

**Keywords:** Venerische Infektionen, Darmkeime, Zikavirus, Ebolavirus, Lymphogranuloma venereum, Venereal diseases, Enteropathogenic microbes, Zika virus, Ebola virus, Lymphogranuloma venereum

## Abstract

Noch vor 50 Jahren waren nur wenige bakterielle Infektionen bekannt, die durch direkten Sexualkontakt auf den Partner oder die Partnerin übertragen werden. Neben der Syphilis und Gonorrhoe wurden der weiche Schanker (Ulcus molle) und das Lymphogranuloma venereum in der Gruppe der klassischen Geschlechtskrankheiten zusammengefasst. Die Infektionszahlen der klassischen Venerea haben am Ende des letzten Jahrhunderts durch die Gefahr einer tödlichen HIV-Infektion abgenommen, sind aber nach der Einführung der hochaktiven antiretroviralen Therapie (HAART) erneut rapide angestiegen und stellen weiterhin trotz erfolgreicher Therapiemöglichkeiten ein nicht zu unterschätzendes Gesundheitsrisiko dar. Durch moderne molekularbiologische Nachweisverfahren, „contact tracing“ und epidemiologische Studien ist nun bekannt, dass die Zahl jener Infektionen, die durch engen Kontakt übertragen werden, wesentlich größer ist. In die Gruppe der „sexually transmitted infections“ werden u. a. neben bakteriellen Infektionen wie Chlamydia trachomatis und Mycoplasma genitalium auch virale Infekte wie Herpes-simplex-, Hepatitis‑B- und humane Papillomviren zusammengefasst. Für einige Erreger, wie das Zika- und Ebolavirus, die Hepatitis A, für gewisse Darmkeime oder die Meningokokken stellt der Sexualkontakt nur einen der möglichen Übertragungswege dar, sie werden „sexually transmissible infections” genannt. Diese Erkenntnisse tragen dazu bei, dass die Bedeutsamkeit von „contact tracing“, einer genauen Diagnostik sowie sexueller Abstinenz über einen gewissen Zeitraum eine wichtige Rolle für die Prophylaxe darstellt, um eine Infektion der zahlreichen Mikroben auf den Sexualpartner zu verhindern.

## Abstract (en)

About 50 years ago, only venereal diseases were known to be exclusively transmitted by sexual contact, such as gonorrhea, syphilis, chancroid, and lymphogranuloma venereum. The number of infections decreased during the end of the twentieth century due to human immunodeficiency virus (HIV), which was associated with a very high risk of death after becoming infected by HIV-positive sexual contact. However, with the introduction of highly active antiretroviral therapy (HAART), the number of infections rapidly increased. Classical venereal infections continue to represent a global health threat. Because of the diagnostic improvement by the introduction of molecularbiological methods and epidemiological studies, it is known that the number of sexually transmitted infections has increased. Chlamydia trachomatis, Mycoplasma genitalium, and viral infections such as herpes simplex, hepatitis B and human papilloma viruses are listed as “sexually transmitted infections” (STI). For some microbes, such as Zika virus, Ebola virus, bacteria of the gastrointestinal area and meningococcal infections, sexual contact is only one possible transmission route of the infection to the partner. They are summarized as “sexually transmissible infections”. The knowledge of infections transmitted to the partner, including contact tracing and diagnostic management plays an important role for the prophylaxis in order to avoid the transmission to the sexual partner.

Die epidemiologische Situation der sexuell übertragbaren Erreger hat sich seit Beginn der 1990er Jahre wesentlich geändert. Einflüsse wie „social networking“, häufiger Partnerwechsel, die Dauer der Infektiosität, globale Reisetätigkeit, Migration, der unterschiedliche Zugang zu Test- und Behandlungsmöglichkeiten sowie das große Testangebot von Amplifizierungsverfahren haben wesentlich zu einem geänderten Spektrum der STIs („sexually transmitted infections“) beigetragen.

Die klassischen Venerea (Syphilis und Gonorrhoe) und bekannten genitalen Kontaktinfektionen, wie *Chlamydia trachomatis* oder der genitale Herpes und die genitalen Papillomvirusinfektionen haben in den letzten Dekaden seit der Einführung von HAART bei HIV-Infektionen zugenommen. Neue Erreger sind in die Liste der STIs aufgenommen worden (Tab. 1).

**Table 1:** 

*1. Klassische Venerea*
Syphilis
Gonorrhoe
Ulcus molle
Lymphogranuloma venereum
Donovanosis
*2. „Sexually transmitted infections“*
Chlamydia trachomatis
Mykoplasmen
Herpes-simplex-Virus
Humane Papillomviren
Hepatitis-B-Virus
Humanes Immundefizienz-Virus
Trichomonas vaginalis
Pediculosis pubis
*3. „Sexually transmissible infections“*
Scabies
Enteropathogene Bakterien
Anaerobe Bakterien
Aerobe Bakterien
Hepatitis-A-Virus
Ebolavirus
Zikavirus
Sprosspilze

Sie werden neben den klassischen Venerea und den „sexually transmitted infections“ (STIs) der Gruppe der „sexually transmissible infections“ zugeordnet. Zu jenen Erregern werden enteritische Keime (Shigellen, Samonellen, Campylobacter, Hepatitis-A-Viren) gezählt, die auch, aber nicht nur durch genitale Praktiken übertragen werden und vor allem bei STI-Risikopersonen eine Rolle spielen. Weiter können bestimmte Bakterien, wie etwa *Neisseria meningitidis (N. meningitidis*), oder bestimmte Viren, wie das Ebola- oder Zikavirus, auch durch sexuellen Kontakt übertragen werden, wenngleich dieser nicht den Hauptübertragungsweg darstellt. Die Zunahme von Resistenzen auf empfohlene Antibiotika, wie sie bei *Mycoplasma genitalium* (*M. genitalium*) oder *Neisseria gonorrhoeae* (*N. gonorrhoeae*) gehäuft auftreten, lassen eine Zunahme von nicht behandelbaren Infektionen befürchten.

## Sexuell übertragbare Keime im Darmbereich

Das Auftreten verschiedener Keime im Darmbereich ist abhängig von deren biologischen Eigenschaften sowie Wirts- und Umweltfaktoren. Häufig, aber nicht ausschließlich treten sie als Ursache des sog. Gay-Bowel-Syndroms bei MSM („men having sex with men“) auf. Die sexuelle Transmission von Darmkeimen kann direkt (oral-anal) oder indirekt (durch fäkalkontaminierte Finger oder Gegenstände) erfolgen. Die Diagnostik basiert auf Kulturnachweis, möglichen PCR-Verfahren oder Toxin-Tests. Ein Gesundheitscheck sollte auch andere sexuell übertragbare Erreger – insbesondere auch HIV – beinhalten. Eine gute Aufklärung hinsichtlich einer Prävention ist essenziell.

### Shigellen

Ihr Name geht auf den japanischen Erstbeschreiber zurück, den Mikrobiologen Kiyoshi Shiga, der bei Menschen und höheren Primatenarten einen sehr direkten oral-analen Kontakt als Hauptinfektionsroute der isolierten Bakterien nachgewiesen hat. Sie sind eng verwandt mit *Escherichia coli* und zählen zu den Enterobacteriaceae. *Shigella sonnei* (*S. sonnei*) und *S. flexneri* wurden in urbanen Regionen isoliert, insbesondere *S. flexneri* mit einem rasanten Anstieg vor allem bei MSM.

Die starke Zunahme dieser Darmkeime ist auf kondomfreien ungeschützten Kontakt mit multiplen Partnern, Chemsex und Antibiotikaresistenz – gegen Azithromycin – zurückzuführen. Die Subtypen S. sonnei biotyp g, S. flexneri subspecies 2a und 3a verursachen speziell bei MSM Entzündungen der Kolonmukosa mit selbstlimitierten Enteritiden bis zu schweren dysenterischen blutigen Darminfektionen, bei denen das Shiga-Toxin eine wesentliche Rolle spielt. Wichtig sind die Abklärung einer Antibiotikaresistenz und die anschließende Einleitung der entsprechenden Antibiotikatherapie.

### Salmonellen

Die meisten humanpathogenen Salmonellenisolate werden der Subspecies *S. enterica* zugeordnet; in den USA konnte man bei 50 % aller Isolate entweder *Salmonella typhimurium* oder *S. enteritidis* nachweisen. Neben Speisen – insbesondere Eiprodukten – kann auch ein direkter oral-analer Mensch-zu-Mensch Kontakt vorwiegend bei MSM entsprechende klinische Symptome eines Gay-Bowel-Syndroms auslösen.

### Campylobacter

Die häufigste Spezies der Campylobactergruppe ist *C. jejuni*, der in vielen Isolaten von kontaminierten Speisen nachweisbar ist. Gemeinsam mit „campylobacter-like organisms“ (CLO) kann er auch bei MSM mit Gay-Bowel-Syndrom isoliert werden. Die klinischen Symptome ähneln jenen einer Shigelleninfektion, wobei Enterotoxine die typischen Krankheitszeichen verursachen. Eine Antibiotikatherapie sollte nur bei Personen mit schweren abdominellen Symptomen initiiert werden.

### Hepatitis A (HAV)

Die Hepatitis A kann ebenfalls orofäkal übertragen werden und verläuft in weniger als 0,5 % der Fälle als schwere Hepatitis. Ähnlich wie bei den bakteriellen enteropathogenen Infektionen stellen MSM die Hauptrisikogruppe dar. Eine große HAV-Epidemie mit 1400 Fällen im Zeitraum von einem Jahr wurde durch Genotypisierung bei MSM in Europa bestätigt, aber auch durch rasche Ausbreitung in Lateinamerika, in den USA und in Südostasien weiterverfolgt.

Eine HAV-Impfung als Prophylaxe ist bei Risikopersonen unbedingt empfehlenswert

Parallel dazu waren hohe Infektionsraten mit HIV und anderen STIs festzustellen. Eine HAV-Impfung als Prophylaxe ist unbedingt bei Risikopersonen empfehlenswert. Auch eine Postexpositionsprophylaxe mit Immunglobulin ist anzuraten.

## Neue sexuell übertragbare Erreger

### Neisseria meningitidis

*N. meningitidis* wird bei etwa 10 % der gesunden Bevölkerung im Nasopharyngealraum nachgewiesen, kann aber auch von anderen Schleimhäuten wie etwa aus dem Urethral‑, Rektal- und Zervikalbereich isoliert werden und klinische Symptome einer Gonorrhoe imitieren. Bei STI-Risikopersonen liegt die Nachweisrate wesentlich höher.

So war bei MSM in über 50 % eine Meningokokkenbesiedelung des Oropharynx nachweisbar, bei heterosexuellen Männern lediglich in 19 %. Bei MSM waren in der Urethra 15 % der gramnegativen Diplokokken als* N meningitidis* identifizierbar, bei heterosexuellen Männern war dies nur in 4 % möglich. Die meisten Isolate waren den Serogruppen B, C, X, und Y zuzuordnen.

Eine Infektion mit *N. meningitidis* ist nur kulturell nachweisbar und wird daher leicht übersehen

Seit 2015 wird in den USA auch bei heterosexuellen Männern mit orogenitalen Sexualpraktiken ein Anstieg der Meningokokkenurethritis beobachtet. Urethritis-assoziierte Meningokokkenisolate besitzen aufgrund des Fehlens von Kapselgenen keine Bakterienkapsel und sind phänotypisch *N. gonorrhoeae* ähnlicher. Unabhängig von dem Auftreten einer Meningokokken-assoziierten Urethritis wurden in den USA in New York, Los Angeles und in Chicago, aber auch in Italien sog. Cluster von Meningokokkeninfektionen durch die Serogruppen C, der hypervirulenten CC11-Gruppe, bei MSM beobachtet, die eine invasive Infektion entwickelten. Eine HIV-Infektion scheint hier einen Risikofaktor darzustellen. Es muss darauf hingewiesen werden, dass eine Infektion mit *N. meningitidis* nicht mittels Amplifizierungsverfahren für *N. gonorrhoeae,* sondern nur kulturell nachweisbar ist und daher leicht übersehen werden kann.

Hinsichtlich der Therapie von genitalen oder symptomatischen oropharyngealen Infektionen mit *N. meningitidis* gelten dieselben Empfehlungen wie für Infektionen mit *N. gonorrhoeae* (siehe Therapieleitlinien der ÖGSTD).

### Ebolavirus

Das Ebolavirus kann über lange Zeit – durchschnittlich 158 Tage – in der Samenflüssigkeit von Überlebenden, sog. Survivors, mittels reverser Transcriptase-PCR (RT-PCR) nachgewiesen werden. Dies bedeutet nicht unbedingt, dass auch tatsächlich infektiöses Virus übertragen werden kann. Allerdings gibt es einzelne Berichte, dass das Virus in der Samenflüssigkeit eines Überlebenden mit dem aus dem Blut der an Ebola verstorbenen Sexualpartnerin isolierten Virusstamm ident war.

Dies bestätigt die Möglichkeit der sexuellen Transmission des Ebolavirus bis zu 179 Tage nach Krankheitsausbruch. Es wird daher derzeit von der WHO ein kostenfreier RT-PCR-Test aus der Samenflüssigkeit von männlichen Überlebenden 3 Monate nach Krankheitsausbruch angeboten und sexuelle Abstinenz bis zu wiederholt negativem PCR-Ergebnis empfohlen.

### Zikavirus

Das *Zikavirus* (*ZIKV*) ist der Gruppe der Flaviviren zuzuordnen mit der Mücke Aedes aegypti als Vektor. Es verursacht eine selbstlimitierte Erkrankung mit Fieber, Kopfschmerzen und Arthralgien. Bereits 2008 wurde von einer Ehefrau berichtet, die gemeinsam mit ihrem Mann nach dessen alleiniger Senegalreise an einer Zika-Infektion erkrankte. Eine Übertragung mit einer Mücke konnte hier ausgeschlossen werden. Dieser erste Verdacht einer sexuellen Übertragung wurde 2013 durch den Nachweis des Virus in der Samenflüssigkeit und im Harn von erkrankten Männern und im Vaginalsekret infizierter Frauen bestätigt.

Obwohl die Übertragung des ZIKV durch die Mücke die Hauptübertragungsart darstellt, empfiehlt die WHO Sexualabstinenz und Kondomgebrauch bis zu 3 Monate nach Krankheitsausbruch. Unbedingt sollte ein Sexualkontakt mit möglicher nachfolgender Schwangerschaft mindestens 2 Monate nach einer Exposition vermieden werden. Weiters sollte während der kompletten Schwangerschaft Kondomschutz oder sexuelle Abstinenz praktiziert werden, sofern eine ZIKV-Transmissionsgefahr besteht.

### Mycoplasma genitalium

**Figure 1:**
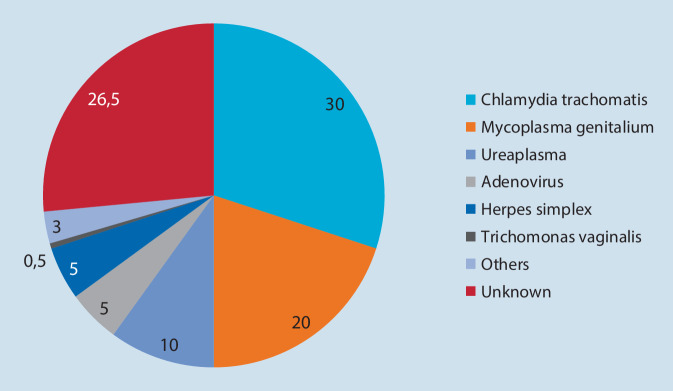

Der Erstnachweis dieser Bakterien gelang bereits 1981 durch D. Taylor Robinson, doch erst seit dem molekularbiologischen Nachweis dieser genitalen Mikroben hat das Wissen über deren Bedeutung zugenommen. Es ist ausreichend bewiesen, dass *M. genitalium* eine nichtgonorrhoische Urethritis (NGU; Abb. 1) und Cervicitis sowie aufsteigende Entzündungen im weiblichen Genitaltrakt verursachen können.

Zahlreiche Studien befassen sich mit der Bedeutsamkeit von *M. genitalium* bei Risikopersonen und bei Individuen mit unterschiedlichen genitalen Symptomen. Der Nachweis mittels PCR wird bei Personen mit genitaler Symptomatik und deren Partnern empfohlen. Die Therapie von *M. genitalium* gestaltet sich aufgrund deren Resistenz auf Azithromycin und Moxifloxacin mitunter schwierig.

Die Makrolidresistenz beruht auf Mutationen im 23S ribosomalen RNA-Gen, jene der Fluorquinolone auf Mutationen in den *parC*- und *gyrA*-Genen. Die höchste Rate wird mit 30–100 % bei MSM angegeben. Diese Beobachtung hat bereits die Empfehlung der Therapie der NGU modifiziert. Es wird Azithromycin über einige Tage empfohlen, eventuell gefolgt von Moxifloxacin (siehe Therapieempfehlung für STI auf der Homepage www.pilzambulatorium.at). Eine Makrolidresistenz kann – allerdings mit gewissem Aufwand – bereits molekularbiologisch abgeklärt werden.

### Lymphogranuloma venereum (LGV)

**Figure 2:**
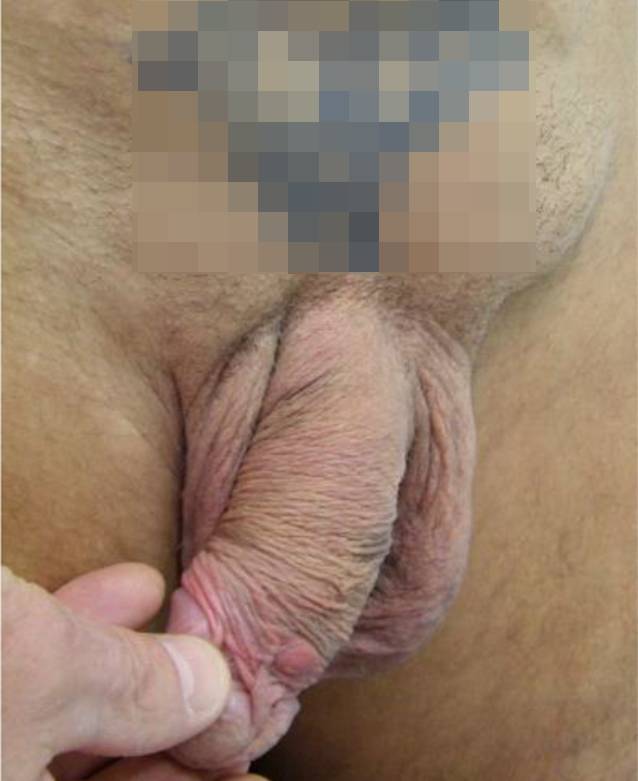

Das Lymphogranuloma venereum (LGV) wird durch LGV Serovar L1–L3 von *C. trachomatis* hervorgerufen und verursacht im Gegensatz zu einer genitalen Infektion mit den Serotypen D–K eine lokale Ulzeration an der Eintrittsstelle mit regionaler Lymphadenopathie (Abb. 2). Eine rektale LGV-Infektion kann eine schmerzhafte Proktitis oder Proktokolitis auslösen.

Seit 2003 haben LGV-Infektionen vorwiegend bei MSM mit rektalen Symptomen zugenommen, meist hervorgerufen durch die LGV2b-Variante und häufig als Koinfektion mit HIV. Die Therapie einer LGV-Infektion mit Doxycyclin wird im Gegensatz zu einer Non-LGV-Chlamydieninfektion mit 21 Tagen wesentlich länger empfohlen. Im Wiener Pilzambulatorium waren 47 % der rektalen Chlamydieninfektionen auf eine LGV-Infektion zurückzuführen. Besonders häufig ist die Koinfektion von rektaler LGV- und einer HIV-Infektion. Personen mit einer rektalen Chlamydieninfektion sollten sowohl auf HIV als auch auf andere STIs untersucht werden.

## Hot topics klassischer Venerea

### Syphilis

Die Syphilis wird von der WHO als ein wichtiges globales Gesundheitsproblem gelistet. Es werden jährlich über 6 Mio. Neuinfektionen weltweit gemeldet. Die letzten Jahre hat die Inzidenz der Syphilis insbesondere bei MSM zugenommen und wird in den USA mit 18,7 infizierten MSM/100.000 Personen im Jahr 2018 im Vergleich zu 11,7 im Jahr 2014 angegeben.

**Figure 3:**
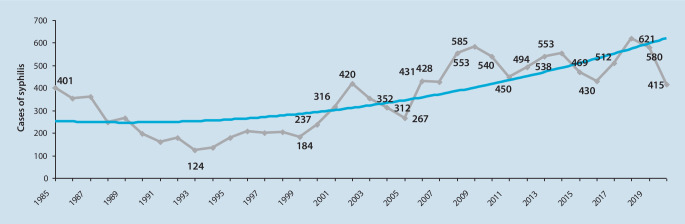

Die in Österreich gemeldeten Zahlen sind in Abb. 3 dargestellt. Während der Pandemie hat die Zahl der gemeldeten Syphilisfälle abgenommen, allerdings muss mit einer nicht unerheblichen Dunkelziffer während des Lockdowns der COVID-19-Pandemie gerechnet werden. Eine Zunahme der Syphilis in den letzten Jahren ist nicht nur bei MSM mit PREP(„preexposure prophylaxis“)-Einnahme, sondern auch bei heterosexuellen Personen zu verzeichnen, allerdings mit ungleich geringerer Zuwachsrate. Die Therapieempfehlung der Syphilis hat sich in den letzten Jahrzehnten nicht geändert; nach wie vor stellt Benzathinpenicillin die Goldstandardtherapie dar, Resistenzen sind bisher nicht beschrieben.

### M. Neisser

Die WHO und CDC (Centers for Disease Control) in den USA haben die Resistenzproblematik von* N. gonorrhoeae* zu einem wichtigen weltweiten Gesundheitsproblem erklärt. Es wird angenommen, dass in den USA etwa 550.000 antibiotikaresistente Gonokokkenstämme zirkulieren. Allerdings werden, im Gegensatz zu Europa und anderen Regionen der Welt, in den USA neben Amplifizierungsuntersuchungen selten Kulturen *von N. gonorrhoeae* angelegt.

Das Gonokokkenresistenzproblem zeigt den Bedarf neuer Antibiotika auf

Seit 2018 zirkuliert sporadisch immer wieder ein Ceftriaxon-resistenter Klon (FC428) in verschiedenen Teilen der Welt. 2018 wurden erstmals 3 Fälle von multiresistenten Gonokokkenstämmen (A2543-Klon) in Australien und UK isoliert, die hochresistent auf Cetriaxon und Azithromycin waren. Pharyngeale und rektale Gonokokkkeninfektionen sind häufig asymptomatisch. Insbesondere pharyngeale Infekte können durch die mangelhafte Antibiotikapenetration vermutlich die Entstehung resistenter Stämme fördern. Das Gonokokkenresistenzproblem zeigt den Bedarf neuer Antibiotika auf. Erste Ergebnisse von Studien mit Solithromycin, Zoliflodacin und Gepoditacin sind bereits zugänglich, endgültige Aussagen sind noch ausständig.

Infektionen im Genitalbereich, einem sehr emotional besetzten Teil unseres Körpers, bleiben somit in der Gesamtschau der verschiedenen *alten* und *neuen* Infektionen weiterhin wichtig. Nur die genaue Diagnostik und eine daraus resultierende gezielte Therapie kann zu Beschwerdefreiheit der Patienten, zu einer Reduktion von Langzeit- oder Spätfolgen und einer Verbesserung der epidemiologischen und klinischen Problematik der STIs führen.

